# Low-Income Turkish Mothers’ Conceptions and Experiences of Family Life

**DOI:** 10.3389/fpsyg.2021.756278

**Published:** 2022-02-14

**Authors:** Gizem Erdem, Merve Adli-Isleyen, Nur Baltalarlı, Ezgi Kılıç

**Affiliations:** ^1^Department of Psychology, Koç University, Istanbul, Turkey; ^2^Trubendorffer Mental Health, Tilburg, Netherlands; ^3^Evo Psikoloji, Istanbul, Turkey; ^4^Department of Psychology, Istanbul Bilgi University, Istanbul, Turkey

**Keywords:** normative family processes, family functioning, low-income families, gender, Turkey, independence, interdependence

## Abstract

The current qualitative study explores women’s conceptions of the normative family and their day-to-day family lives. To that aim, we conducted five focus group interviews in two low-income neighborhoods of Istanbul. The sample included 43 women (42 biological mothers and a grandmother) who had at least one child between ages 3 and 8 in their care. Participants were 35.64 years old on average (SD = 4.74) and were all married. Women had approximately two children (SD = 0.72) whose mean age was 7.92 years old (SD = 3.11). Each focus group was semi-structured, lasted for 1–1.5 h, and included 5–12 participants. Thematic analysis of the focus group interview data, moderator memos, and observer’s notes revealed five defining features of healthy family functioning: cohesion, healthy child, parenting, conflict, control, and family organization. Overall, women prioritized motherhood over their other social identities and idealized the happy family, which contradicted their actual lived experiences in the family system. We discuss how women’s depictions of all family processes revolved around cultural constructs of gender, socio-economic status, and independence/interdependence. The findings of this study shed light on future interventions for low-income women and their families in Turkey.

## Introduction

In the past three decades, substantial literature has documented the negative consequences of poverty for child development and family well-being (Brooks-Gunn and Duncan, 1997; Bhana and Bachoo, 2011). Children in poverty are at higher risk of dropping out of school (Chaudry and Wimer, 2016), engaging in delinquency (Ponnet, 2014), and suffering from anxiety and depression (Karevold et al., 2009), than those in affluent families. Current research addresses marital relationships, positive parenting, and responsiveness as protective mechanisms that mediate the relationship between financial strain and child social and emotional development (Harold and Leve, 2019). Some researchers have moved from sole examination of the parent-child relationships among low-income families and focused on the family as a system (e.g., Walsh, 1996, family resilience) and how families cope with adversity. Several key family processes that characterize resilient families are family cohesion, communication, family organization/control and discipline, routines and rituals, and the social support network (Black and Lobo, 2008).

Despite the empirical support for the protective family processes that impede the adverse effects of poverty, there are critical gaps in the literature. Little is known about the parents’ (especially mothers’) ideal standards of family well-being, their day-to-day experiences, and their interactions with other family members. Several studies explored mothers’ child-rearing experiences in the context of mental illness (Funk et al., 2012), homelessness (Labella et al., 2019), and poverty (Son and Bauer, 2010; Sano et al., 2020). However, those studies were restricted to ethnic minority single mothers, most of whom resided in urban cities of Western countries (Roubinov and Boyce, 2017). There is limited research examining the normative family processes of low-income urban families in non-Western countries where families are more likely to be intact (compared to single-mother-headed households in the United States). Turkish families attribute different values to the child and fertility (Kaǧıtçıbas̨ı, 2005) and have close ties to extended family members, kins, and the community. In an attempt to address those gaps, the current study investigates how low-income Turkish mothers with young children in their care idealize and construct a “healthy, normal and happy family.” Our study also explores discrepancies between actual experiences of family life and the ideal and how parents evaluate such family processes and resilience factors.

### Normative Family Processes

According to the Circumplex Model (Olson et al., 1979; Olson, 2000, Olson, 2011), family dynamics and functioning are conceptualized *via* three dimensions; cohesion, flexibility, and communication. Family cohesion refers to the emotional bond and warmth between family members, ranging from disengagement to enmeshment. The second dimension, flexibility, is the family’s capacity to change its leadership, roles, and rules to adapt to the environmental demands, developmental changes, or life events. Flexibility ranges from chaos (complete disorganization) to rigidity (maintaining the *status quo* despite the changing conditions; Olson, 2011). Communication is a facilitative dimension that helps families alter their flexibility and cohesion (Olson, 2011). It is expected that cohesion and flexibility dimensions are curvilinear such that moderate levels of those dimensions reinforce happiness, satisfaction, and high functioning in the family (Olson, 2000).

The Circumplex Model and the hypotheses it generates are supported by numerous research samples from Western countries (Kouneski, 2002; Mirnics et al., 2010). Yet, Olson et al. (2019) acknowledged that many of these studies were conducted in the United States, mainly with Caucasian, Christian, and middle-class families (also known as Western, educated, industrialized, democratic, and rich (WEIRD) samples; Henrich et al., 2010). Despite the model’s universality claim, several cross-cultural studies indicate poor support of the model’s premises (Kouneski, 2002). For instance, research has shown that family processes with high cohesion and high rigidity are valued and encouraged in Asian (Lee, 1996) and Jewish-Orthodox communities (Pirutinsky and Kor, 2013). The current study expands on that knowledge and investigates Turkish mothers’ conceptions of the normative family processes and their experiences.

### Normative Family Processes and Socio-Economic Conditions of the Families

Studies document that low-income families experience higher family conflict, parenting stress, and issues in family functioning due to financial strain (Wadsworth and Compas, 2002; Ponnet, 2014; Botha et al., 2018). A systematic review of family resilience in low and middle-income countries (Bhana and Bachoo, 2011) found that impoverished urban families cope with financial strain *via* a positive outlook in life, a strong sense of purpose, and family cohesion and warmth. In addition, extra-familial support from kins and community ties are vital factors that promote resilience among low-income families.

Families in low-resource settings also adapt their organization to respond to financial struggles. Parents allocate long hours to work, which inevitably limits their time for family leisure and daily routines, such as mealtime, television time, and play (Coyl-Shepherd and Hanlon, 2013; Quintana et al., 2018). Family organization is intertwined with the decision-making process, control, and power in the family. For instance, extended family members in low-income families may relieve the parents’ burden by sharing childcare responsibilities and becoming salient support. Yet, they also participate in the decision-making process regarding the child’s upbringing and interfere with parenting (Sunar, 2002).

In their review, Bhana and Bachoo (2011) found that high parental discipline and emotional expressiveness toward the child predict positive social and academic outcomes for low-income children. While authoritative parenting and high family ideals remain beneficial for low-income families, gender roles in the context of parenting and child care are changing. Research has shown that the percentage of dual-earner couples is increasing among low-income families, and fathers are becoming more involved in child care than before (Raley and Bianchi, 2006; Kenney, 2008; Lück and Ruckdeschel, 2018). Yet, fathers’ involvement is restricted to playing with children rather than providing physical or emotional care (Gracia, 2014; Musick et al., 2016). In a recent study, Crapo et al. (2021) investigated mothers’ and fathers’ expectations and beliefs about parenting in a large sample of parents from different socio-economic status. All parents prioritized children’s independence and believed in the significance of parental discipline. However, mothers prioritized emotionally connecting with their children, whereas fathers emphasized maintaining peace when dealing with children. Those findings indicate how parenting beliefs and practices remain gendered across families of different socio-economic status.

### Research on Meaning of Healthy Family in Turkey

In sum, literature with WEIRD samples emphasizes the cohesion and flexibility dimensions of the family with a focus on their balance and demonstrates the utility of family communication in family functioning. However, a current study in Turkey (Turkdogan et al., 2019) has shown that high cohesion and rigidity are associated with higher marital satisfaction and family functioning. Turkish families are characterized as psychologically interdependent in their structure where family needs and expectancies are prioritized over to individuals’ needs, but autonomy and independence of children are also highly valued (Kaǧıtçıbas̨ı, 1996, 2005; Sunar and Fis̨ek, 2005; Turkdogan et al., 2019). Contrary to the Circumplex Model perspective, those diffused boundaries between family members may not be necessarily associated with family dysfunction or relationship dissatisfaction (Erdem and Safi, 2018). Instead, enmeshment (extreme cohesion) is perceived as the relatedness of family members or an indicator of family support (Turkdogan et al., 2019). Turkish families have gender hierarchy (i.e., men over women) and generational hierarchy (i.e., grandparents over parents; Sunar and Fis̨ek, 2005). Gender hierarchy is associated with strict gender roles, parenting responsibilities, and control over women and children. Women’s perceived gendered role as primary caregivers of children continues regardless of their employment status or education level, indicating low flexibility of family structures in Turkey (Fis̨ek, 1991; Sunar and Fis̨ek, 2005; Turkdogan et al., 2019).

In low-income families, women seek employment to ease off the family’s financial strain. Still, they are expected to take a *second shift* (Hochschild and Machung, 1989) and fulfill parenting tasks and housework. Nationally representative recent studies show that Turkish men have gradually become more engaged in housework and childcare (Zeybekoǧlu, 2013; Bozok, 2018; O’Neil and Çarkoǧlu, 2019). Yet, father involvement with children is restricted to 1–2 h a day on average (S̨ahin et al., 2017) and include playing with children or giving them rides (Bozok, 2018). Besides, men’s involvement in family life is perceived as “helping” or “assisting” women, rather than taking equal responsibility for the tasks as spouses or fathers (Bozok, 2018). Among low-income Turkish families, fathers spend significantly less time with children because they work longer hours and have fewer financial resources to afford extracurricular activities (Biber, 2016).

Besides, working women in Turkish low-income families still lack economic independence and power in financial decision-making processes. Therefore, women’s employment is more of a necessity to make ends meet than a sign of empowerment (Sunar and Fis̨ek, 2005). Working full time is perceived as a distraction from taking care of children; therefore, some low-income women prioritize child care, especially when children are young. A nationally representative study on fatherhood by Mother Child Education Foundation (AÇEV) demonstrated that Turkish men continue to see their role as breadwinners and protectors, consistent with traditional gender roles (Bozok, 2018). Except for parental control, both care and nurturing of children fall on women.

Given that hierarchical structure, mothers become mediators of communication between fathers and the children (Fis̨ek, 1991; Sunar and Fis̨ek, 2005). These gendered roles are internalized and accepted as a norm among Turkish women and are maintained in low-income and rural families (Baydar et al., 2010; Özdemir et al., 2020). Nevertheless, current studies suggest that young married couples are more egalitarian in the division of housework and childcare approach between the couple, especially in urban areas (Zeybekoǧlu, 2013; Bozok, 2018; Bayer, 2020).

Another aspect of normative family structure in Turkey is the meaning attributed to having children. According to the nationally representative Turkish Institute of Statistics Family Structure Surveys (TURKSTAT, 2017), 99.7% of married adults reported that their primary motive for getting married was to rear children. Studies document that Turkish couples attribute *a psychological value* to having children (Kaǧıtçıbas̨ı and Ataca, 2005). That is, couples cite the joy of raising children and building a meaningful emotional connection with them as the primary motives to become parents. Such attributions of urban modern Turkish families indicate a shift from social or economic reasons to have children (e.g., child contributing to the family income, family reputation) in the past three decades. Bayer (2020) argues that parents’ expectations from their children have also increased as fertility rates decreased nationwide. Thus, parents dedicate their resources to fewer children but have higher hopes for and expectations from their children.

### The Current Study

Turkish families have undergone substantial changes in marriage, divorce, and gender roles in the past decade. Consistent with the demographic trends worldwide, fertility rates are decreasing in Turkey, whereas divorce rates, women’s employment and education level, and age at first marriage are rising (TURKSTAT, 2020). Recent community surveys have shown that Turkish urban, middle income and educated couples have more egalitarian values and attitudes in marriage (Zeybekoǧlu, 2013; Bozok, 2018; Bayer, 2020) while low-income rural families continue to be more traditional (Aytaç, 1998; Aykan and Wolf, 2000). Nevertheless, Turkish families also have distinctive characteristics. Research suggests that Turkish families are highly cohesive, inflexible in gender roles, and hierarchical (Turkdogan et al., 2019).

Additionally, the nature of the communication in the family is often non-verbal, and mothers act as mediators in the family to manage conflict and disclosure (Gulerce, 2007). The Turkish case is unique with contradictory properties – family demographics show a rapid change in family structure, but hierarchy seems stable. The experiences of low-income families may fall in either of those extremes in the continuum; less is known about the characteristics of low-income families who have traditional ties to their hometowns but are urbanized and acculturated in liberal attitudes of family life. The current study addresses this gap in the literature.

## Materials and Methods

### Participants and Setting

We recruited participants through elementary schools from two low-income disadvantaged neighborhoods in Istanbul. Blinded to the study’s aim and scope, school counselors informed parents about the project through face-to-face communication, e-mails, and flyers. To be eligible, women had to be (a) at least 18 years old, (b) a parent or a primary caregiver of at least one child from 3 to 8 years old, and (c) a native Turkish speaker. To prevent contamination of research findings, we excluded the parents who were currently participating in the ongoing parent training seminars in schools. All participants who met the eligibility criteria were invited to the study. We collected data from December 2018 to April 2019.

The final data included a convenience sample of 42 biological mothers and a grandmother. The sample size was determined based on data saturation. That is, similar themes emerged across interviews. In addition, the number of focus groups matched the suggested standards for qualitative studies (Braun et al., 2019). The age of participants ranged from 28 to 50 years old (Mean = 35.64, SD = 4.74). All participants were currently married, and the average duration of marriage was 13.9 (SD = 5.88) years. Participants had approximately two children on average (SD = 0.72) whose mean age was 7.92 years old (SD = 3.11). The sample was economically disadvantaged. Of 43 primary caregivers, 12.5% reported having a monthly cost of living under 1.600 TL (below minimum monthly wage in Turkey during data collection, equivalent to ∼117 USD per person). Additionally, 40% of the sample had a monthly cost of living ranging from 1.600 TL to 3.000 TL, whereas 32.5% had 3.000 TL to 4.500 TL monthly expenses. Only 15% of the parents had monthly living costs higher than 4.500 TL, which corresponds to the official poverty level across the nation (∼329 USD). The socio-demographic characteristics of the participants are presented in Table 1.

**TABLE 1:** Socio-demographic characteristics of participants (*N* = 43).

	Mean (SD)	*f* (%)
Age	34.64 (4.74)	
Duration of marriage	13.9 (5.88)	
# of children	2.02 (0.72)	
# of boys	1.22 (0.79)	
# of girls	1.0 (0.735)	
Age of children	7.92 (3.11)	
Monthly household expenses*		
Under 1,600 TL		5 (12.5%)
1,600 – 3,000 TL		16 (40.0%)
3,001- 4,500 TL		13 (32.5%)
higher than 4,500 TL		6 (15%)

*TL refers to Turkish Lira.

*The minimum monthly wage was 1,600 TL (∼117 USD) per person during data collection.*

*According to Turkish Statistics Institute estimates, the official poverty level for a family was at 4,500 TL (∼329 USD) monthly.*

*Data were missing for three participants.*

*The table shows valid percentages.*

### Procedure

The ethics committee of the Koç University approved all study procedures (Protocol no: 2019.065.IRB3.040). We sought official permission from the school principals and the related district’s municipality following IRB approval. We collaborated with school counselors to announce the study and recruit participants. Women who met the eligibility criteria signed up for the focus groups. On the day of focus groups, the research team welcomed the participants, introduced themselves (credentials, research goals, and clinical expertise), described the study procedures, and obtained written consent. Participants filled out a brief demographic form and continued with the focus group. All interviews were conducted in a conference room of the selected school. School counselors were not present at the meetings.

The study utilized a qualitative research methodology to gain an in-depth understanding of women’s experiences of family life and conceptions of a healthy family. We preferred the focus groups over in-depth individual interviews because they enabled us to detect the consensus and divergence among the participants (Morgan, 1996) regarding family relationships. The focus groups were semi-structured with questions and probes about normative family processes and family functioning (see Table 2 for the protocol and questions). A facilitator (a clinical psychology graduate student) and a notetaker (an undergraduate research assistant) moderated each focus group. All facilitators (*n* = 3) were Turkish women and were 24–26 years old. Through their graduate coursework, facilitators were trained in clinical interviewing skills and qualitative research methodology. Facilitators followed the study protocol and led the group discussion while the undergraduate research assistants observed participants’ interactions and took notes. The research team had no prior relationship with either the participants or the school counselors. They had, however, previous research experience in couple and family relationships in Turkey.

**TABLE 2:** Focus group protocol and semi-structured interview questions.

*Process:* A facilitator and notetaker welcome participants, introduce themselves, and explain their roles and the group procedures. After the consent form is presented and signed, they continue with the protocol below. *Introduction protocol:* “We have organized this meeting to gain an understanding about your experiences as parents/primary caregivers. All personal information you disclose here today will be kept strictly confidential and will not be shared with the school staff or family members with any identifiers.”
1. What are the characteristics of a healthy and happy family? (If not specified, probe with the following questions: How do you define healthy relationships in a happy family? From whom does the family get support in difficult times? How do you define roles and rules?) 2. What adjectives would you pick to describe a healthy and happy family? 3. How would you describe healthy child development? Which traits, characteristics, and behaviors show that a child is developing healthily? 4. In your opinion, what kind of events or situations could negatively impact the well-being of your child and your family? What do you think will do the most harm? (Follow-up question to expand the discussion: What worries you most about your family and child?)

All interviews were digitally recorded. There were five focus groups in total, and group sizes varied from 5 to 12 participants. Each meeting took approximately 1–1.5 h to complete. Participants were compensated for their time with the option to attend a parenting seminar. After each focus group interview was complete, facilitators wrote field notes. Both observers and facilitators independently sent their notes to the study PI (the corresponding author) within a week.

### Data Analysis

After completing the data collection, the digital recordings of focus groups were transcribed verbatim. Qualitative data also included the field notes such as note takers’ observation notes, facilitators’ field notes, and reflections. The study’s goals were to capture how low-income women characterize an ideal and functioning family and explore their day-to-day family lives. Therefore, we were interested in understanding participants’ explicit and implicit ideas around the normative family and exploring common patterns across data. We selected the Thematic Analysis method (TA; Braun et al., 2019) for data analysis because it fit our study goals to explore, describe, and comprehend participants’ experiences, mindsets, and realities. TA organizes and summarizes datasets in rich detail, combining obtained information under meaningful themes. Among different approaches to TA and conceptualization of themes, we used the “shared-meaning based patterns” approach (Braun et al., 2019), which explores themes that organize findings around core concepts, ideas, and interpretations that are common across the interviews. This approach is contrary to the “domain summary analysis,” which is based on participants’ responses to a particular question and analyzes the responses at a surface level of meaning. Because we were interested in more overarching themes that were repeated and were salient in the interviews, we approached the data analysis with a focus on themes across different focus groups rather than answers to particular questions.

Research assistants (RAs) independently coded the transcripts under the supervision of the principal investigator (PI), a Turkish clinician, and an experienced researcher who was a novice to the content of the focus group meetings. Of five coders, three were graduate students in a clinical psychology program and were also facilitators of the focus groups. Two other coders (an undergraduate and a graduate student) were psychology students blinded to the research project’s objectives. We did not use qualitative software to manage or code transcripts.

We followed Braun and Clarke (2006) six steps of thematic analysis. Initially, RAs read both transcriptions and field notes several times to familiarize themselves with the complete data and have a general understanding of the participants’ perspectives. Second, PI selected a specific focus group interview which RAs independently coded and identified unique features of the participants’ perspectives. During the coding process, RAs produced as many codes as possible to capture the details of the participants’ experiences. Each RA developed its list of codes with a label/name, a definition, and exemplar quotes. Next, RAs and PI read all code lists and met to discuss potential areas of disagreement in coding and revised the codebook. After there was a consensus on codes and identifying them, RAs continued with coding for the rest of the interviews. Next, codes were gathered together and collated under potential themes. RAs checked whether themes matched the assigned data extract and the entire data. We created visual tools, such as maps, to simplify themes and sub-themes during this phase. The themes were clearly described and labeled. We finalized the themes based on their relevance to the research question and the participants’ reports by the total agreement of the entire research team. We checked data extracts, memos, and field notes to ensure that final themes adequately captured repeated meanings. In the last step, the final themes were reported by consensus. In sum, we followed an inductive data analysis procedure which included open coding, creating themes, and abstraction. This is contrary to a deductive TA that involves data coding as an exemplification of predetermined themes.

Our data analysis process follows a “coding reliability approach” (also called *consensus coding*; Braun et al., 2019). Our approach to coding is based on agreement among multiple coders, aims for singular and a shared analysis of qualitative data, and is guided by a codebook. We acknowledge that our coding reliability approach prioritizes research rigor (especially consistency across coders) from a post-positivist stance. That is, we used qualitative research as a tool and a technique rather than a research paradigm in the current study.

### Trustworthiness

We utilized various strategies in data collection, data analysis, and reporting of results to enhance the study’s trustworthiness (qualitative research rigor). Elo et al. (2014) argue that indicators of trustworthiness are credibility, dependability, conformability, and authenticity. To capture more information, we used multiple data collection methods (data triangulation), including digital audio recordings and field notes. Triangulation enhanced the credibility of the findings. The experiences of research participants were described accurately and adequately *via* multiple sources of data. We collected data from different schools and compared and contrasted focus group interviews to ensure dependability (data stability over time and under different conditions). In addition, we gathered codes and themes by the consensus of all research team members to avoid potential bias or motivation of a single researcher in interpreting the findings. Developing a codebook ensured consistency of the results across coders and allowed for the potential replicability of the study by other researchers. To prevent the contamination of the data analysis, three graduate students involved in the focus groups did not code the transcript of the session they facilitated. Instead, they coded the transcript that was completely unfamiliar to them. Besides, we had novice researchers (another graduate student and an undergraduate student) involved in the data analysis process. These research procedures increased the conformability of the study (the potential for congruence between two or more independent coders about the data’s accuracy, relevance, or meaning). Lastly, we reported general themes across interviews, exceptions, and contrary perspectives of participants. This approach increased the authenticity of the study (the extent to which, as researchers, we are fair and faithful to the participants to represent a range of realities). The PI oversaw the entire data collection, analysis, and reporting of findings to strengthen the overall reliability of the current work. Research design, data analysis, interpretation, and reporting followed the Consolidated Criteria for Reporting Qualitative Studies (COREQ; Tong et al., 2007) guidelines.

## Results

### Family Processes and Structure: Women’s Experiences in and Conceptions of the Family

#### Cohesion

Women emphasized that a healthy family should actively build and promote a sense of togetherness. In addition, cohesion constituted enjoying quality time with each other, having mutual respect and trust, and feeling appreciated and accepted by other family members. Participants argued that those characteristics were ideal for a closely-knit, healthy family (Table 3).

**TABLE 3:** Summary of findings: Women’s conceptions of the family by cultural constructs.

		Cultural constructs		
		Gender roles	Socio-economic status	Independence/interdependence
Conceptions of the family	Cohesion	Mutual respect and trust, supportive; women are selfless, loving, and affectionate.	Nuclear family, caring for one household	A sense of togetherness and love; affection for children, respect among the couple
		Women are drivers of cohesion.		Cohesion centers around the parent-child relationship, rather than the couple
		Women negotiate, compromise and sacrifice.		
	Child as subsystem (healthy child)	No gendered expectations from children	Children are expected to survive rather than thrive	Expectations from children: morality, obedience, respect (Both autonomy and relatedness)
		Women are at the center of ensuring child’s happiness and well-being	Fear, worry about potential harm to children (sexual abuse/bullying)	Caring for the children is the hallmark of family life
			Mistrust anyone outside the nuclear family	
	Parental subsystem	Mother = compassionate, primary caregiver; Father = disciplined, sets rules, meets material needs, low involvement.	Men work long hours to make ends meet, have no time for childcare, and have few hours at home.	“Our father” rather than a spouse
		Trying hard not to replicate own parents; fear of making mistakes and being an inadequate mother.		
	Conflict	When things go wrong, it is the mother’s fault.	Need financial assistance and help with childcare, seek support from in-laws	Marital conflict over parenting styles and child discipline; disagreements should be kept private (away from children); in-laws may intervene.
	Control and family organization	Family rituals and daily practices to welcome the father in the evening.	Difficulties in setting boundaries with in-laws	Both gender hierarchy and generational hierarchy are present.
		Men and his family have control and power over the family.		Parental control and warmth are intertwined.

Love and affection were also associated with family cohesion, but they were defined differently for the parent-child relationships and marital relationships. Women perceived love as a form of care for children, and early childhood memories influenced their conceptions. One mother stated, “My dad didn’t love us back in the days. He didn’t express his love [and he was] too cold and distant. I want my daughter to experience love to its fullest. The subject of love is a profoundly precious one for me.” (Focus Group 2, ID 17).

Regarding marital relationships, women emphasized support, sacrifice, and loyalty as signs of love. The majority of the women voiced the importance of giving up on their own needs for the sake of other family members. One woman stated that “My husband spent 10 days in the hospital. I did not leave him for a minute, I didn’t even go home and take a bath” (Focus Group 3, ID 24). Another participant explained how women were drivers of cohesion in the family and should understand men. “Not only children but also men seek happiness in the family. They work all day, are stressed out. On top of that, if we [women] complain and tell them this and that about children, there will not be any peace in the family. Only when women welcome their men with a smile at the door can men provide love and care to the family” (Focus Group 1, ID 1).

Cohesion also included emotional and instrumental support of extended family members. A mother described that “if we face problems, we consult our grandparents, our elders for advice, what to do” (Focus Group 4, ID 28) and expressed gratitude and respect for financial and childcare support.

#### Healthy Child: Child as a Subsystem

The third question of the interview protocol was related to definitions of a healthy child. Yet, participants discussed that theme throughout the interviews, including their depictions of the family unit as whole. Overall, the focus group interviews revealed that participants perceived the ideal family as child-centered, where the ultimate goal was to ensure the developing child’s well-being and happiness (Table 3). Therefore, the family was built and organized around the needs of the children. Besides, a healthy child was characterized by good physical health and desirable personality traits (a sense of confidence, obedience, responsibility, and an easy temperament). Several participants underscored the importance of assertiveness and good communication skills. A mother described the healthy children as someone who can “easily speak up their mind whether the mothers are present or not” (Focus Group 4, ID 37).

Notwithstanding the importance of confidence, several participants emphasized that children should comply with social norms while asserting themselves. A mother stated, “of course, the optimal way is [my child communicates] without making a scene and attacking others” (Focus Group 5, ID 38). Participants noted that being responsible and well-behaved were interrelated: children should be held accountable for their actions, be conscious of their environment, and pay attention to the demands of the family. Additionally, several participants expected their children to differentiate good from evil and recognize the possible troublesome situations. Thus, expectations of morality and obedience co-existed with expectations of the child to be assertive to protect oneself from bullying. One woman said, “I may be mistaken…I mean, I probably am. I tell my children” “do not harm anyone, but if a friend pushes you or attacks you, defend yourself” (Focus Group 4, ID 33). It appears that mothers wanted their children to comply with the family rules but be autonomous enough to protect themselves when needed.

#### Parenting: Conceptions of Motherhood and Fatherhood

In general, women frequently referred to the parent-child and parental subsystem as core features of a normative family. Nevertheless, women rarely discussed the couple’s relationship when describing the normative family. When few participants mentioned the importance of the couple’s relationship, they discussed it as an alliance to perform parenting tasks and responsibilities for the child’s well-being and happiness. One woman explained, “you should spend quality time with children, as a couple (…) Father should show affection to the mother when children are present [in that way] children learn to be loving” (Focus Group 1, ID 10; same theme in Focus Group 2, ID 20).

Women differentiated the roles in the family by gender. One woman stated, “men should work, and women should attend to [care for] their children” (Focus Group 4, ID 28). Women wanted men to be more involved in parenting, but the majority empathized with men about difficulties in doing so. Several participants explained that men work long hours and need time to rest (e.g., Focus group 1, ID 1 and 10) or men simply did not know how to show affection due to their upbringing (e.g., Focus group 1, ID 2). On the other hand, women’s responsibilities were expansive, regardless of their employment status. Women in focus groups took care of household chores and children while mediating daily interactions among the family members. One of the participants said it is a task, rather than a choice: “women are always doing this mothering thing, they are fulfilling their duties. But fathers… they do it only if they want to do it” (Focus Group 1, ID 9). Women’s responsibilities to give care were not only physical but also psychological. One participant stated, “As a mother, my happiness equals the family’s happiness, I need to have some peace of mind to keep peace at home” (Focus Group 4, ID 31). This theme appeared in other groups as well (e.g., Focus Group 1, ID 1; Focus Group 2, ID 16 and 18).

While there was a consensus among women about feeling exhausted and overwhelmed with parenting responsibilities, their attitudes toward gender inequality differed. Some women believed these gendered parenting roles were the norm and fundamental to family life. Others argued that these roles were unfair but felt hopeless about changing them. For instance, in Focus Group 5, three participants said that “we are born that way.” One woman explained further; “all of us, be it adults, or children… I think we all have a nature, like a disposition. They cannot change. One should not push too hard to change that. For instance, I would never expect my spouse to clean the windows. He did not grow up in that way.” A similar discussion occurred in Focus Group 1, where ID 3 discussed how she enjoyed equal division of domestic and childcare labor at home (an exception throughout the focus groups). Other women were astonished that she “could trust men to cook, clean or even care for children.” The general theme was that men and women had different lives and dispositions.

Motherhood was also associated with mixed emotions; there was a confusion over being a good enough parent, anxiety over protecting the child, and mistrust of external family members and the community. Because women perceived themselves as the primary caregivers, they felt full responsibility for protecting children from harm. Women were confused about how to ensure safety but not intimidate their children. One mother said, “My kids [have] never asked such questions [about self-protection] to me. But when they do, I don’t want to misinform them. If they ask, what should I tell them? I want to make sure I can give short and clear answers” (Focus group 1, ID 2). Another mother expressed a dilemma: “some people may harm my children, it is harmful if they touch my children [refers to sexual abuse]. We can warn our children and tell them three times in a month, but then when they go outside or are at school, they will fear ‘something will happen to me’ then they would not want to go to school. They will think something bad will happen, they will say ‘someone will hurt me.’ We need to warn them without worrying them too much. Otherwise, [children] will be too scared” (Focus Group 2, ID 13). One participant expressed her fears as, “We see and hear these on TV so often. Everyone outside is dangerous (…) We are a nuclear family. I feel like everyone outside of our house is a potential threat” (Focus Group 4, ID 36).

There were mixed opinions about working mothers and how to balance family and work life. A participant voiced, “in fact, mothers should work. But, when children are young, mothers should be around. Even if mothers work, they should not be too busy. For instance, they should arrange work hours accordingly” (Focus Group 4, ID 33). Another mother expressed concerns over employment and care, reflecting on her own childhood experiences. “I believe mothers, including myself, should not work because when I was young, my mom was working. I am kind of sensitive about this. I prefer having issues to make ends meet, but be there for them. Children should see their mothers at the door welcoming them upon return from school so that they know she cares” (Focus Group 4, ID 28).

#### Conflict

Participants endorsed the ability to manage conflict as a characteristic of a normative family. Women explained that conflict occurred over parenting, child discipline, or financial issues. This theme intersected with co-parenting to a great extent. For instance, one woman reported that “If I say no to my child, my husband should say no too. If not, we get in fights” (Focus Group 2, ID 16). Also, another participant articulated how her parenting style was different from her spouse’s “My husband is very fond of our daughters. When I set boundaries, he worries that their hearts are broken, and it is my fault” (Focus Group 5, ID 41). Women believed that parents should manage conflict effectively and keep it private in an ideal family. A mother stated that “sometimes we argue and fight and we reflect it to the child. It is inevitable; we don’t know what else to do” (Focus Group 3, ID 24). Mothers also reported conflict with in-laws, especially over boundaries and childcare.

#### Control and Family Organization: Boundaries

Women emphasized the importance of family rules and rituals, such as enjoying dinner, removing electronics during meals, and scheduling play and TV time. Those practices were also linked to setting boundaries and discipline (Table 3). A participant explained how the family rituals and daily routines represented respect for the father: “Every evening when *our father* comes from work, we line up at the door and welcome him home” (Focus Group 4, ID 31). This ritual indicated a depiction of a family organization; there was a gender hierarchy between spouses and a generational hierarchy between father and children. A few women desired a more democratic parent-child relationship and argued that children should participate in the decision-making process in the family (e.g., Focus Group 2, ID 18).

Women expressed difficulties in setting rules and boundaries with their children. They were struggling to balance parental warmth and control. Some women shared their fear of losing connection to their children when they set too much discipline and control. Another dilemma was the ambiguous boundaries between the married couple vs. in-laws. Most women explained how parenting styles differed among family members, which was confusing for children. Some participants indicated that they wanted to prevent in-laws from intervening in childcare and discipline as much as possible. Therefore, only when they faced intense financial strain or emotional difficulties could they seek help from extended families. Only a few mothers stated they would consult a mother-in-law or father-in-law for advice or support. One woman said, “freedom is an important issue; we do not have the freedom to parent as we want because we have mothers-in-laws” (Focus Group 2, ID 16). Her experience is yet another example of the gender and generational hierarchy within the family system.

### Comparisons Across Focus Groups: Notes and Observations

Several similarities and differences across focus group interviews are noteworthy. Overall, women agreed that mothers are the primary caregivers, and their well-being is directly linked to family functioning. Additionally, all women discussed the immense domestic labor and childcare tasks they had to fulfill, complained about the limited father involvement, and expressed exhaustion and anxiety over being good enough parents. However, women differed in their attributions of inequality in their family lives. Inequalities were attributed to sex differences between men and women and their dispositions, societal expectations, psychological factors (e.g., men’s childhood experiences), or financial insecurity and strain. Only a few women endorsed egalitarian attitudes in child-rearing or parenting.

Throughout the interviews, women focused on the parent subsystem and parent-child subsystem rather than the spousal subsystem. Although the first two interview questions did not probe their roles as mothers, they were inclined toward that direction, revealing how the imagery of a healthy family centered on the developing child and was secured by the mother. The language women used to describe their family lives and conceptions supported that claim. Several women referred to their spouses as “our father,” “family’s father,” or “the husband,” rather than “my husband” (e.g., Focus Group 3, ID 22). Only three women (those who endorsed egalitarian values) carefully and consistently referred to men as “my spouse.” A similar theme was evident when women were discussing *love*^1^ Throughout the interviews, none of the participants brought up romantic love (*as̨k*) to their spouses or discussed time alone with their partners as part of rituals. Instead, they debated love around compassion, affection, trust, and respect by referring to it as *sevgi*.

Besides, focus groups were heterogeneous regarding participants’ attitudes and values. For instance, in Focus groups 1 and 4, participants disagreed about their expectations from fathers (whether or not men are competent enough to take care of children) and mothers (whether or not women with children should work). One exception was Focus Group 3, which had relatively fewer participants, more engagement in the conversation, and more agreements, indicating a groupthink process. Finally, we observed that women sought validation from moderators often. Women raised questions about their parenting practices in focus groups 1, 3, 4, and 5. They either asked if they were “doing the right thing” or asked what they should do instead. In focus group 2, women rarely asked questions, but they gave examples of their parenting and waited for moderators to reflect. Such a quest for validation could be related to the professional identity of the moderators (who were clinical psychologists, hence, perceived as experts on the family matters) or the pressure women felt as primary caregivers (the idealized motherhood and associated responsibilities).

## Discussion

The current study explored low-income women’s conceptions of normative family processes and their actual day-to-day family lives. The study’s findings suggested that women characterized healthy family functioning and processes around cohesion, healthy child, parenting, conflict, control/family organization. It was evident that women idealized motherhood in every aspect of their lives and gave examples of sacrifice. Idealized mother kept the solidarity of the family with compassion (family cohesion), was the primary caregiver for children (parenting), ensured moral and social development of the children (healthy child), and accepted and enforced obedience to the father while bridging the relationship between him and the children (family organization/control). In sum, women’s conceptions of the family were related to Olson (2011) Circumplex Model and three domains of family functioning; cohesion, flexibility, and communication. However, as hypothesized, definitions, interpretations, and connotations of those processes went above and beyond the Circumplex Model and were embedded in the culture. We argue that women’s family conceptions revolved around cultural constructs of gender, socio-economic status, and independence/interdependence. Our findings and discussion points are summarized in Table 3.

### The Conception of the Family and Its Subsystems

A notable finding of our study relates to the lack of emphasis on the couple subsystem and marital relationship, even in idealized family narratives. Women referred to healthy family functioning as building connections to the kin (e.g., children, own parents, and in-laws), but not their spouses. This construction of family contrasts a relatively narrow depiction of the family as a nuclear household with a mating unit at the center. Shweder et al. (1995)’s principle of the sacred couple suggests an understanding of family life around the couple’s emotional intimacy, commitment, and sexual privacy. Such family conceptions are typical of WEIRD families (and their high independence), which disproportionately inform current family science research and theory. Our study revealed that Turkish women prioritized parent-child relationships over marital relationships and built a complex web of relationships with their families-of-origin and in-laws.

Boratav et al. (2014) argue that such interdependent depictions of the family also point to the resistance to egalitarian gender role ideologies and the construction of masculinity in Turkey. A hierarchical understanding of gender promotes the development of mutual respect and tolerance between the couple over building emotional intimacy or having direct communication. Therefore, women may seek closeness and support elsewhere - typically through relations to their children or kins, and the conception of the family shifts its focus on parenting and child well-being. Indeed, participants in our study referred to motherhood as an emotionally fulfilling experience. Still, motherhood was also a duty rather than a choice.

### Cultural Constructs of Cohesion, Control, and Parenting

Family processes related to cohesion, control, and parenting practices were defined around traditional gender roles. Those findings are consistent with previous research findings in Turkey (Sunar and Fis̨ek, 2005; Bayraktar, 2011; Boratav et al., 2014; Bozok, 2018). Other existing literature shows that, despite the ever-increasing awareness of gender equality, parenting roles remain gendered, especially in families with low-education backgrounds (Musick et al., 2016; Crapo et al., 2021). Even in dual-income families, women perform a disproportionate amount of childcare, domestic labor and meet the psychological needs of all family members (Jolly et al., 2014; Negraia et al., 2018). In the current study, women were also responsible for preserving and continuing the family organization, and they complained about the unequal distribution of domestic labor. It appears that gender roles as norms continue for low-income women, even those conscious of such inequalities (Baydar et al., 2010; Özdemir et al., 2020). Economic restrictions may also intensify gender inequalities in parenting. Studies in Turkey document that low-income fathers carry an intense workload (compared to middle-class fathers), and their long working hours hinder their involvement in child care (Biber, 2016; Bozok, 2018). Hence, women’s reports of the ideal family structure are indicators of traditional gender roles, which are further reinforced with their socio-economic circumstances.

Meanings associated with family cohesion and control were also linked to the cultural constructs of independence/interdependence. Women defined cohesion (closeness, sense of togetherness, love), control, and organization (discipline, rules, rituals) as interrelated to one another. Consistent with a cultural model of interdependence, both processes ensured family unity and harmony. As Kaǧıtçıbas̨ı and Sunar (1992) argued, the presence of control did not connote a lack of love in Turkish families. Instead, control was essential for the child’s moral development and was necessary to protect the family organization and family members’ well-being. Women defined the family by high intimacy, low flexibility, indirect communication, and diffused boundaries (a definition that fits The Circumplex Model’s *enmeshment* category). However, parallel with the previous research on the Turkish family (Sunar and Fis̨ek, 2005), this particular combination of intimacy and hierarchy was the norm among Turkish families.

### Balancing Autonomy and Relatedness in the Family in Parental Subsystem

It is important to note that interdependence and independence may co-exist within cultures (Raeff, 2010), and cultural practices of interdependence vary across so-called collectivistic cultures (Harkness and Super, 2002; Kaǧıtçıbas̨ı, 2012). For instance, women in our sample sought extended family support (typically grandmothers) in childcare and family finances as needed. However, instrumental support came with a cost, with older women in the extended family (especially mothers-in-law) taking control over younger women (especially brides), interfering with parenting tasks and responsibilities and violating the boundaries and perceived freedom. This finding exemplifies how interdependence and independence are compatible cultural aspects of child-rearing and other practices in the families.

A similar process was evident in women’s desire to show affection and love toward their children. As the value of children shifted to psychological (rather than economic or utilitarian) value, mothers have started to encourage the expression of emotions. Sunar (2002) argued that such changes could be attributed to urban women’s divergence from harsh parenting due to the fear of disengagement from their children. While these trends do not appear to have reduced emotional closeness within the family, they constitute a potential individualizing trend in future generations, promoting higher autonomy of children. In the meantime, women seem to fulfill their perceived primary role as mothers by performing the ideal mother with compassion and openness in emotional expression.

An additional example of co-occurring independence and interdependence was related to women’s ways of communicating their needs. Women defined their needs as identical to the needs of their children and the family. In an ethnographic study with low-income mothers, Bayraktar (2011) found that women perceived their children almost like an organic part of themselves. She further argued that such identification allowed women to indirectly voice their concerns, desires, and needs. Similarly, women in our sample raised issues in family life *via* conversations around child development or parenting rather than their emotional or social needs. That is, women’s emphasis on connection to their children (needs for relatedness) was linked to interdependence in the family and their need for autonomy.

### Expectations From Children

One common expectation women had from their children was obedience, a finding consistent with prior studies with Turkish samples (Sunar, 2002). A national survey of parents indicated that child’s disobedience to the parents and disrespect to the elderly were the most unacceptable misbehaviors (TURKSTAT, 2017). Consistent with family models of psychological interdependence, urban Turkish parents restrict children’s autonomy while encouraging conformity and dependency (Sunar and Fis̨ek, 2005). Parents punish a child’s anger or disobedience toward authority to reinstate their power (Sunar, 2002).

Women sought confidence and assertiveness from their children as well. Those expectations from children can be interpreted through cultural constructs of interdependence, gender, and socio-economic status. From a cultural perspective, the transition in the value of children and rising urbanization in Turkey can indicate changes in parental expectations from children (Kaǧıtçıbas̨ı and Ataca, 2005). Istanbul attracted migrants from rural areas, which resulted in a rapid change in parenting attitudes (Sunar and Fis̨ek, 2005). Parents adopted a nuanced stance to child-rearing; they promoted the development of self-confidence and autonomy, but they also expected obedience to secure psychological interdependence and relatedness (Kaǧıtçıbas̨ı, 2005). From a gender perspective, our findings relate to the hierarchical formulation of the family roles and structure itself. Men were portrayed as patriarchs with the power to rule and make decisions, which inevitably placed women and children in a position of obedience. On the other hand, women did not expect complete obedience from their children. Kandiyoti (1988) argued that obedience is associated with femininity in Turkey and represents passivity, inadequacy, and weakness. Therefore, women in the current study may have desired some confidence and assertiveness from their children to demonstrate strength.

Last but not least, women’s expectations from their children (and their parenting practices) could be related to their socio-economic status. Overall, women were apprehensive about the safety of their children. Women wanted to rear assertive children so that children could protect themselves from abuse and bullying. Kusserow (1999) found that low-income parents from unsafe neighborhoods wanted to prepare their children for life’s hardships, expected them to “stand up for themselves” or “speak their minds.” In that way, children would acquire the necessary skills to survive in a harsh environment. Kusserow’s (1999) concept of *hard defensive individualism* was evident in women’s rhetoric in our sample. Similar to the ethnic minority working-class parents, women in our study expected obedience from their children and emphasized respect and hierarchy in the family. Yet, assertiveness meant promoting the autonomy of children as a defense against life struggles. Of note, those expectations are quite contrary to those of middle-class parents who do not have safety concerns for their children. Instead, middle-class parents see children as unique individuals who should have the freedom to explore life and reach their potential (defined as *soft offensive individualism*, Kusserow, 1999). Our findings demonstrated that independence/interdependence could have different meanings and practices for low-income mothers.

### Limitations and Strengths of the Study

The findings of our study should be interpreted with caution due to several methodological limitations. Our recruitment sites were located in two low-income urban neighborhoods. Working mothers or families residing in other low-income areas across Istanbul or other cities in Turkey may have significantly different family life experiences, cultural practices, and conceptions. In addition, all women participating in the study were recruited through schools. Hence, there is a possibility that they were already primed to consider their roles as parents. We also acknowledge that we did not involve participants in data analysis and interpretation, as suggested by COREQ (Tong et al., 2007). For instance, we did not return the transcripts to the participants for correction or request feedback on the findings. Those issues reflect a limitation in the validity of our results. Finally, using focus groups rather than in-depth individual interviews may have triggered groupthink processes where participants could be inclined toward consensus. Individual interviews could allow for more diverse opinions and experiences to be expressed.

Despite those limitations, our study contributes to the literature in several ways. Our study focuses on the unique experiences of Turkish women and highlights the nuances in their family conceptions. While women internalized gender roles, they were conscious of gender inequality in their family lives. On the one hand, women were recipients and enforcers of authority (to seek obedience); on the other hand, they desired freedom for themselves and their children. Our study adds to the current literature by showing that family life and conceptions in Turkish families are complex and multifaceted.

### Implications for Future Research

Our study findings illustrate a need to understand women’s conceptions of the family as a cultural construct with three main components: gender, socio-economic status, and interdependence/independence. More research is needed to unfold the interplay between those cultural components, family practices, and functioning. For example, parents’ expectations for their children’s independence and interdependence change as children develop (Raeff, 2010) and vary by gender and socio-economic status (Harkness and Super, 2002). Because culture is a dynamic process, future research could investigate how parenting practices, family rituals, and child well-being change over time. Such research will help us capture potential differences in family conceptions and changes in gender roles and financial status. For instance, research on the value of children suggests an increasing number of urban families (regardless of their socio-economic status) prefer having a girl, rather than a boy in Turkey (Kaǧıtçıbas̨ı and Ataca, 2005). More research is needed to understand whether family conceptions (cohesion, organization, healthy child) in the current study are outcomes associated with changing values attributed to children and sex preference of a child in Turkey.

Additionally, our study focused solely on women. More research is needed to examine how the social roles of mothers and fathers relate to each other and are influenced by cultural practices. Future studies could investigate whether women’s depictions of motherhood change when men get involved in child care. It is unclear if internalized gender norms refrain women from seeking more father involvement as they feel pressured to fulfill the role of a perfect mother. Prior research indicates that mothers compensate for the lack of father involvement through sharing childcare with older women in the extended family (Can, 2019). Future research could investigate how women negotiate and share those responsibilities and how solidarity re-shapes or reinforces family processes and structure among Turkish families.

Finally, it was evident that women in our study were overwhelmed with domestic work, parenting stress, and the psychological labor to bring the family together. More research is needed to explore the direct and indirect costs of family life for low-income Turkish women, both physically and mentally. An accumulation of research demonstrates that underprivileged minority adults (including those with financial strain) experience chronic stress associated with a higher allostatic load and, therefore, higher proneness to physical and mental health problems (Beckie, 2012). It is worth exploring whether low-income Turkish women experience similar psychological and physical health concerns in the long term.

## Conclusion

Our research indicated that women’s conceptions of family processes did not simply show their values and attitudes toward the family or their family lives. Instead, those family conceptions revealed how family processes were understood, valued, and structured around gender roles, socio-economic status, and independence/interdependence. Thus, cultural constructs co-exist, grow, and change within and across family systems and influence current child-rearing practices and family development. Ultimately, addressing how those cultural constructs are re-shaped within the family (and how gender roles and independence/interdependence are perceived) can add to our understanding of the complexities of culture and family development.

## Data Availability Statement

The raw data supporting the conclusions of this article will be made available by the authors upon request.

## Ethics Statement

The studies involving human participants were reviewed and approved by Koç University Social Sciences Ethics Committee. The patients/participants provided their written informed consent to participate in this study.

## Author Contributions

GE designed the study, oversaw data collection, consulted data analysis, co-wrote sections of the manuscript, and edited the full manuscript. MA-I collected and analyzed data and drafted several sections of the manuscript. NB collected and analyzed data, assisted MA-I in writing the results section and preparation of tables. EK collected and analyzed data, assisted MA-I and NB in writing the results section and discussion of the manuscript. All authors contributed to the article and approved the submitted version.

## Conflict of Interest

The authors declare that the research was conducted in the absence of any commercial or financial relationships that could be construed as a potential conflict of interest.

## Publisher’s Note

All claims expressed in this article are solely those of the authors and do not necessarily represent those of their affiliated organizations, or those of the publisher, the editors and the reviewers. Any product that may be evaluated in this article, or claim that may be made by its manufacturer, is not guaranteed or endorsed by the publisher.

## Footnotes

1 In Turkish, there are different words to refer to love: *As̨k* indicates an intense emotion that is romantic and passionate. Folk stories and songs refer to it as “merging with the other” or “burning together with desire.” *Sevgi* is an emotion that makes the person feel closer, connected, and committed to the other. It has connotations with peace and a sense of togetherness. In the current sample, women endorsed *sevgi* as a building block of the family.
